# Mesenchymal Stem Cells Therapies on Fibrotic Heart Diseases

**DOI:** 10.3390/ijms22147447

**Published:** 2021-07-12

**Authors:** Fernanda Gubert, Jaqueline Soares da Silva, Juliana F. Vasques, Renata Guedes de Jesus Gonçalves, Robertta Silva Martins, Mauro Paes Leme de Sá, Rosalia Mendez-Otero, Gisele Zapata-Sudo

**Affiliations:** 1 Instituto de Ciências Biomédicas, Universidade Federal do Rio de Janeiro, Rio de Janeiro 21944-590, Brazil; fernanda.gubert@icb.ufrj.br; 2 Programa Redes de Pesquisa em Saúde no Estado do Rio de Janeiro, Rio de Janeiro 21944-590, Brazil; julianavasques@biof.ufrj.br (J.F.V.); renata.guedes@biof.ufrj.br (R.G.d.J.G.); rmartins@biof.ufrj.br (R.S.M.); 3 Programa de Pesquisa em Desenvolvimento de Fármacos, Instituto de Ciências Biomédicas, Universidade Federal do Rio de Janeiro, Rio de Janeiro 21944-590, Brazil; ssjck@hotmail.com; 4 Instituto de Biofísica Carlos Chagas Filho, Universidade Federal do Rio de Janeiro, Rio de Janeiro 21944-590, Brazil; 5 Instituto do Coração Edson Saad, Faculdade de Medicina, Universidade Federal do Rio de Janeiro, Rio de Janeiro 21944-590, Brazil; mauropaesleme.cirurgia@gmail.com

**Keywords:** mesenchymal stem cells, cardiac fibrosis, cardiovascular diseases

## Abstract

Stem cell therapy is a promising alternative approach to heart diseases. The most prevalent source of multipotent stem cells, usually called somatic or adult stem cells (mesenchymal stromal/stem cells, MSCs) used in clinical trials is bone marrow (BM-MSCs), adipose tissue (AT-MSCs), umbilical cord (UC-MSCs) and placenta. Therapeutic use of MSCs in cardiovascular diseases is based on the benefits in reducing cardiac fibrosis and inflammation that compose the cardiac remodeling responsible for the maintenance of normal function, something which may end up causing progressive and irreversible dysfunction. Many factors lead to cardiac fibrosis and failure, and an effective therapy is lacking to reverse or attenuate this condition. Different approaches have been shown to be promising in surpassing the poor survival of transplanted cells in cardiac tissue to provide cardioprotection and prevent cardiac remodeling. This review includes the description of pre-clinical and clinical investigation of the therapeutic potential of MSCs in improving ventricular dysfunction consequent to diverse cardiac diseases.

## 1. Introduction

In recent decades, stem cell therapy has emerged as a promising alternative approach to several chronic or acute pathological conditions, including heart diseases. Among the different types of stem cells studied for their therapeutic potential, the main types include: 1. embryonic stem cells (ESCs), obtained from the blastocyst, which can give rise to many cells in the body, except for the placenta and umbilical cord; 2. induced pluripotent stem cells (iPSC), derived from skin, blood or other somatic cells, which are genetically reprogrammed to behave like an ESC; 3. multipotent stem cells, usually called somatic or adult stem cells (such as mesenchymal stromal/stem cells, MSCs), which are more specialized than ESCs, and are important to maintain and repair different tissues [1]. MSCs are one of the most used cell types in clinical research. Several characteristics favor their use in a wide range of diseases, such as their capacity to home and respond to a damaged host environment, releasing factors that could act in different pathways protecting and/or inducing the regeneration of injured tissue, the minimal ethical restrictions and to their safety immunological profile [2]. The potential therapeutic use for this cell type was reinforced after the authorization granted for Osiris Therapeutics in Canada (2012) [3], and JCR Pharmaceuticals in Japan (2015) [4] for the treatment of acute graft-versus-host disease. Stem cell therapy, including iPSC and resident progenitor/stem cells such as MSCs, has been widely evaluated for the treatment of heart diseases [5]. iPSCs have the potential to differentiate into cardiomyocytes (“cardiac regeneration”), but it has been shown, in pre-clinical models, to induce teratoma formation [5], and inadequate maturation and integration of derived cardiomyocytes could induce arrhythmias after transplantation [6]. Although adult stem cells/progenitors rarely differentiate into cardiomyocytes after transplantation, they induce myocardial repair due to their paracrine effects. A wide range of ischemic or non-ischemic heart diseases could benefit from MSCs therapeutic potential, making them one of the most promising cell types for heart therapy among the many types of adult progenitors/stem cells tested until now [7].

## 2. MSCs Sources for Therapy in Heart Diseases

The most prevalent source of MSCs evaluated in clinical trials from different pathological conditions is bone marrow (BM-MSCs), followed by adipose tissue (AT-MSCs), umbilical cord (UC-MSCs) and placenta [4]. The therapeutic action of BM-MSCs is likely related to secretion of growth factors, cytokines, chemokines, microRNAs and extracellular vesicles (EV), which attenuate inflammation and cardiac remodeling, improving neovascular formation and inducing endogenous cardiac regeneration [7]. The secretoma of BM-MSCs has immunosuppressive properties through the modulation of immune cells including dendritic cells [8]. AT-MSCs have numerous advantages compared to BM-MSCs: more abundant and affordable source; the initial yield is higher and cell senescence is reduced. As BM-MSCs, the main mechanism of action of AT-MSCs is through their paracrine effect, which is related to a prominent angiogenic effect [9]. It was also described that the immunomodulatory capacity of AT-MSCs may be higher than that of BM-MSCs [10]. However, it is important to emphasize that although the collection of adipose tissue is minimally invasive, there is a risk of pulmonary venous embolism and injury to other organs and sepsis [11]. Similar to BM-MSCs, the reduced quality of cells obtained from aged donors or from patients with important comorbidities, such as diabetes, are a concern for the use of autologous AT-MSCs. In this regard, the umbilical cord (UC) is an interesting source of young MSCs, without the need of an invasive biopsy and minimum ethical concerns, as this tissue is usually discarded after birth. In addition, they have greater proliferative capacity even after cryopreservation and cellular senescence is reduced compared to BM-MSCs and AT-MSCs, due to a more primitive phenotype, longer telomeres and more active telomerase [12,13]. Furthermore, allogeneic transplantation does not imply a higher risk of rejection due to the low immunogenicity of MSCs, which lack MHC II and co-stimulating antigens CD80 and CD86 expression, as well as an immunosuppressive secretoma [14].

Herein, we provide an overview of the current research on the therapeutic application of MSCs in several cardiomyopathies, highlighting the benefits of cell therapy in cardiac fibrosis and inflammation.

## 3. Signaling Pathways Related to Cardiac Fibrosis

Cardiac fibrosis is a pathophysiological disorder commonly associated with acute and chronic cardiovascular diseases, such as myocardial disease, aortic stenosis, arrhythmia, diastolic and systolic dysfunction, cardiac insufficiency, coronary diseases and others [15,16,17,18,19]. Cardiac remodeling is important to maintain cardiac function based on molecular, cellular and interstitial changes in the heart. However, if stressful events are sustained, remodeling may end up causing a progressive and irreversible dysfunction, resulting in an excessive extracellular matrix (ECM) deposition and induction of pro-fibrotic events [20,21]. The cardiac ECM is mainly composed of fibrillar forming collagen (types I and III), but it can also be rich in other non-fibrillar components such as type IV collagen, glycosaminoglycans, glycoproteins, proteoglycans, fibrin, fibronectin, elastin and laminin [22,23,24]. ECM is also a reservoir of growth factors, cytokines, chemokines, proteases and their inhibitors and microRNAs [22], which play important roles in fibroblasts proliferation, migration and transdifferentiation.

Cardiac fibroblasts interact with cardiomyocytes directly and via paracrine signaling [25,26,27] because they synthesize and secrete ECM components, such as collagen, fibronectin and proteoglycans as well as ECM-degrading matrix metalloproteinases (MMPs). Fibroblasts play a key role in normal ECM turnover, which is essential for the structural integrity of myocardial tissue. During the fibrotic processes, pathological deposition of ECM occurs due to proliferation and transdifferentiation of fibroblasts into activated myofibroblasts [15,28]. Myofibroblasts start to express alpha smooth muscle actin (αSMA), incorporated into newly formed contractile fibers, which confers cellular contractility to facilitate tissue repair and increase the secretion of ECM proteins [29,30,31]. Fibroblast transdifferentiation is induced by mechanical stimuli consequent to increased hemodynamic overload and damaged cells and by molecular mediators, such as angiotensin, aldosterone, cytokines and growth factors secreted by inflammatory cells and cardiomyocytes [15,16,32].

Signaling pathway of transforming growth factor-beta (TGF-β1) is one of the main mechanisms related to the induction of cardiac fibrosis [33,34,35]. In cardiomyocytes and ECM of normal adult hearts, a high expression of inactive TGF-β1 is observed. However, after the occurrence of heart damage, active TGF-β1 dimers are released and may regulate the phenotype and function of different cell types, especially the fibroblasts involved in tissue repair. TGF-β1 induces myofibroblasts conversion, which, in addition to ECM proteins, also produces hypertrophic and pro-fibrotic factors [29,36,37]. The TGF-β1 signaling pathway leads to phosphorylation of Smad2 and Smad3 proteins when linked to Smad4 translocate to the nucleus to induce the transcription of target genes [34,38,39]. TGF-β1 signaling can also follow non-canonical pathways with the involvement of mitogen-activated protein kinase (MAPK), p38 MAPK, c- Jun N-terminal kinase (JNK 1/2) pathway, phosphoinositide 3-kinases and protein kinase B (PI3K/AKT), extracellular signal-regulated kinases (ERK1/2) [32,40,41], as well as AKT and glycogen synthase kinase 3 (AKT/GSK3β) [26,42]. All of these proteins play an important role in fibrotic responses by TGF-β1. Angiotensin II (Ang-II) may act by modulating TGF-β1 though the activation of the TGF-β1/Smad pathway, leading to the differentiation of fibroblasts into myofibroblasts. Ang-II is elevated in patients with cardiovascular diseases, something which has been associated with the pro-fibrotic condition. A blockade of the activated renin-angiotensin system interposes the fibrotic remodeling [43,44,45]. Pro-fibrotic activity of TGF-β1 can also be regulated by aldosterone through the activation of mineralocorticoid receptors or indirectly mediated by its interaction with Ang-II [46,47,48].

Inflammation plays an important role in cardiovascular diseases, and pro-inflammatory cytokines can be associated with cardiac fibrotic events. Tumor necrosis factor alpha (TNFα) and interleukins 1 and 6 (IL-1 and IL-6) have their levels increased during cardiac injury and are involved in tissue remodeling [49,50,51,52]. Increased TNFα response is related to cardiac fibrosis while the inhibition of this cytokine attenuates fibrotic events [51,53]. TNFα has two receptors responsible for mediating its responses, TNFR1, associated with deleterious effects and TNFR2, related to protective profile [49,54]. Ang-II can induce TNFα synthesis and synergistic signaling between Ang-II and TNFα, through the activation of TNFR1 results in an excessive collagen synthesis responsible for cardiac fibrosis [54,55]. Furthermore, TNFα is also linked to cardiac fibroblasts proliferation and fibronectin deposition, increased activity of MMP9, and differentiation of cardiac fibroblasts into pathogenic myofibroblasts [14,55].

The cytokine IL-1 promotes the production of MMP, contributing to ECM remodeling and cardiac fibrosis with increased collagen production by myofibroblasts [52,56,57]. IL-1 stimulates IL-6, a pro-fibrotic factor that triggers TGF-β /Smad signaling [49,58]. Additionally, the upregulation of the JAK2/STAT3 pathway seems to be involved in the cardiac hypertrophic effect of IL-6 [50,51]. In contrast, IL-10 is an example of a well-characterized anti-inflammatory cytokine in the heart, considered a therapeutic strategy for cardiac fibrosis. IL-10 inhibits TNFα-induced apoptosis, reduces hypertrophy induced by pressure overload or by Ang-II, improves cardiac function and attenuates cardiac remodeling [51].

MMPs are extracellular matrix proteolytic enzymes responsible for the degradation of several ECM macromolecules. Dysregulation of their functions can lead to excessive matrix deposition, which facilitates cardiac fibrosis [59]. MMP-2 and MMP-9 are the most abundant in cardiac tissue and are considered biomarkers of cardiac fibrosis [60,61]. MMP-9 is implicated in the activation of TGF-β, release of TNF-α, cleavage of IL-1β, migration of cardiac fibroblasts and collagen production [60,62,63]. MMP-2 has been shown to play a protective role preventing cardiac remodeling, but controversially seems to be associated with fibrosis and an increase in TGF-β signaling [20,60]. MMP-3 contributes to cardiac fibrosis because it can be upregulated by increased Ang-II during cardiac remodeling. MMP-3 can alter fibroblast viability, migration and apoptosis. It has been associated with regulation of angiogenesis and is involved in ECM remodeling [64,65].

MicroRNAs are small non-coding RNAs that regulate transcription through post-transcriptional degradation of messenger RNA and can participate in cardiac fibrosis [35,66]. Recently, the opposed roles of microRNAs in fibrotic process were described: miR-433, miR-21, and miR-125b seem to promote cardiac fibrosis, while miR-150, miR-29a, miR-30, miR-133, and miR-590 could suppress the fibrotic response [45,66]. These microRNAs were shown to regulate pathways involved in fibrosis, miR-21 upregulation attenuates Smad7 expression; an inhibitory protein of the TGF-β/Smad signaling pathway [35,67]. In addition to this, positive miR-21 feedback has also been observed in Ang-II -mediated ERK/TGF-β/Smad signaling [35,67]. miR-21 have been reported in patients with aortic stenosis, hypertrophic cardiomyopathy and dilated cardiomyopathy [35,68]. In contrast, miR-132 reduces TGF-β expression after cardiac ischemia [69]. MSCs induce the release of extracellular vesicles (EV) containing microRNA molecules [70].

**Figure 1:**
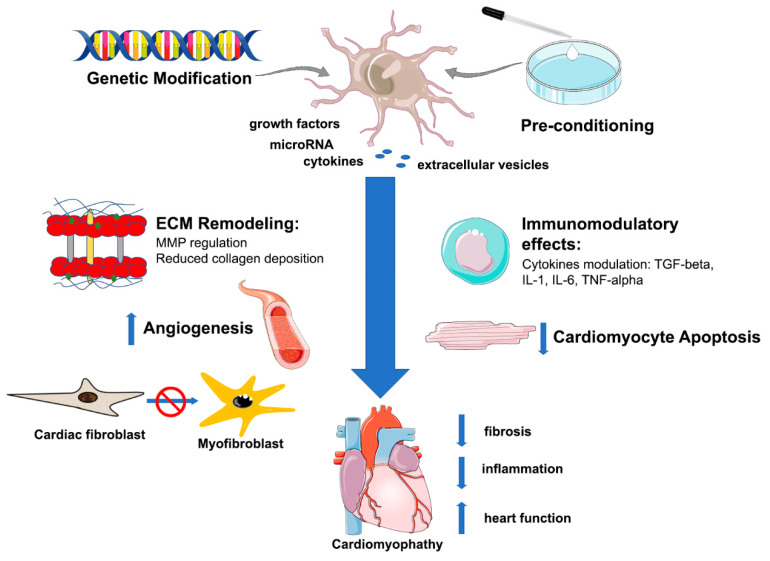
MSCs therapy in cardiomyopathy. MSCs release growth factors, microRNAs and cytokines, directly or in extracellular vesicles, which modulate different aspects of cardiomyopathies. MSCs reduce fibrosis by modulating MMPs, reducing collagen deposition and inhibiting the conversion of cardiac fibroblasts to myofibroblasts. MSCs also reduce inflammation, stimulate angiogenesis and reduce cardiomyocyte apoptosis. All of these factors result in cardiac function improvement, as observed in pre-clinical models. MMP: matrix metalloproteinases. Created using https://smart.servier.com/.

The main factors that lead to cardiac fibrosis are growth factors, inflammatory mediators, interleukins, microRNAs, which increase the synthesis and deposition of ECM elements as well as stimulate cardiac fibroblasts differentiation, resulting in cardiac fibrosis and failure. Although the knowledge of pathways implicated to cardiac fibrosis, still lack effective therapies to reverse or attenuate this condition. MSCs are an alternative to treat cardiac fibrosis in different cardiomyopathies, since they can provide cardioprotection and prevent cardiac remodeling. The signaling pathways related to cardiac fibrosis that are involved in MSCs therapy are shown in Figure 1.

## 4. MSC Therapy in Pre-Clinical Models of Cardiomyopathies

### 4.1. MSC Therapy in Ischemic Cardiomyopathies

Coronary artery diseases are still the main cause of death around the world, and myocardial infarction (MI) induces an extensive remodeling that is associated with an irreversible condition, leading to heart failure (HF). MSCs therapy was tested exhaustively to recover cardiac tissue after acute and chronic MI. Pre-clinical studies showed that MSCs therapy in acute MI with improved cardiac function is safe because it prevented the initial cardiac damage before remodeling. In chronic ischemic disease, MSCs therapy aimed to modulate inflammation and reduce scar size. A meta-analysis including 58 studies showed that MSCs therapy after acute and chronic MI, promoted reduction of infarct size and increase of left ventricular ejection fraction (LVEF), in mice, rats and swine [71]. Considering the immunomodulatory potential of MSCs, these cells were therapeutically tested in a porcine model of microvascular obstruction, it has been demonstrated that MSCs could prevent cardiac fibrosis, increase neovascularization in infarct and border zones and reduce infiltration of inflammatory cells. In addition to this, therapy reduced infarct area and increased LVEF [72].

Although MSCs show promising results to treat HF, transplanted cells have poor survival in cardiac tissue, especially in an ischemic environment, which could compromise their efficacy in clinical studies [73]. As an alternative to improve cell survival, different approaches showed to modify the host environment through the inhibition of local oxidative stress or to interfere with the transplanted cells to tolerate and respond better in a condition of damaged tissue. Genetic manipulation has been a tool to increase MSCs potential and survival. Modified BM-MSCs with increased expression of C1q/TNF-related proteins (CTRP3) increased the cells’ survival from 50 to 85% when injected in the ischemic myocardium. This observation was accompanied by reduced interstitial fibrosis, which was confirmed by low fibroblast density and increased myocyte/fibrotic cell ration [74]. One further option is to increase homing capacity of MSCs by overexpressing integrin-linked kinase (ILK), which showed enhanced homing to infarcted myocardium compared to non-modified MSCs. MSC-ILK attenuated cardiac remodeling, reduced cardiac fibrosis and improved heart function [75,76]. Another approach to improve the therapeutic potential of MSCs is their pre-condition before transplantation. The exposure of murine AT-MSC for 24 h to sphingosine 1-phosphate (S1P), a natural bioactive metabolite, enhanced AT-MSC migration ability to the infarcted area, which provided the reduction of heart remodeling and function after MI [77]. Transplantation of murine BM-MSC pre-treated with asprosin, an adipokine, promoted a reduction in cardiac fibrosis and improvement on LVEF [78]. Both strategies induced enhanced MSCs homing and survival, oxidative injury protection and anti-apoptotic potential in ischemic tissue, possibly by the activation of ERK1/2 and Akt pathways.

Since the infarcted area is a hostile environment to MSCs survival, the combination of cell therapy and pharmacological approaches to alter local tissue was used to improve cell engraftment. Atorvastatin improved MSCs survival and reduced cardiac fibrosis in swine submitted to MI MSCs [79]. Concurrent use of exogenous extracellular matrix (ECM from rabbit heart) and MSC transplantation reduced MSC apoptosis, leading to a reduced infarct area and improved left ventricular function after MI [80]. In an attempt to induce a better outcome after cell therapy, the protein adropin was administered together with the pre-exposed BM-MSC to adropin, but even when not altering MSC survival, this method reduced fibrotic area [81].

An important inconvenience for the clinical use of MSC therapy is the quality pattern of cells and the cost of treatment. Innovative strategies to deliver cells more efficiently have been recently developed and evaluated in MI pre-clinical models. Using spray formulation, cells are uniformly distributed, heart function is improved and cardiac fibrosis attenuated [82,83]. The EV derived from MSCs includes microRNA, mRNAs, growth factors, cytokines and enzymes which could mimic the therapeutic potential of MSCs, modulating inflammation and promoting survival/regeneration of the damage area [84]. The therapeutic benefits of MSC-derived exosomes in cardiac repair and regeneration have been successfully reported [85]. In pre-clinical swine models of chronic MI, myocardial injection of EV derived from human BM-MSCs increased blood-flow in the ischemic region, however, EVs intravenous injection failed [86,87].

Therefore, cardiac fibrosis is an important feature usually analyzed after MSC therapy in models of acute or chronic MI. However, the mechanism by which MSC could act to reduce fibrosis is still under investigation. MSCs administered just after MI induction could prevent cardiomyocytes apoptosis, which ultimately would result in a reduction of regional damage and scar formation. However, there is evidence suggesting a direct action of MSCs to prevent cardiac fibrosis. MSC could release MMP, providing an effect on fibrosis or modulating cardiac fibroblasts to produce less collagen. MSC cultured in conditioned medium decreased viability and collagen production of cardiac fibroblasts [88]. Intraventricular transplantation of MSC, two weeks after MI induced in rats, promoted an increase of heart function and decrease of cardiac fibrosis with less deposition of collagen type I and type II with increased MMP2 and HGF levels [88]. In contrast, when MSCs were injected intravenously, MM9 and MM14 but not MMP2 were increased in heart tissue. The therapeutic potential of MSC in reducing cardiac fibrosis depends on MM9 level, since MSCs derived from MMP-9 knockout mice did not modify fibrosis [89].

### 4.2. MSC Therapy in Non-Ischemic Cardiomyopathies

The therapeutic potential of MSCs in cardiac fibrosis and inflammation has also been investigated in pre-clinical models of non-ischemic cardiomyopathies (NICM), which include heart muscle diseases with highly variable underlying etiologies (idiopathic, genetic, metabolic, infectious) that lead to ventricular dysfunction and HF.

Dilated cardiomyopathy (DCM) is the most common type of NICM and the main cause for heart transplantation. Intramyocardial injection of BM-MSCs attenuated tissue fibrosis and improved cardiac function in a rodent model of DCM induced by immunization with porcine cardiac myosin [90]. The cell transplantation induced angiogenesis and reduced myocardial collagen volume fraction as well as protein content of MMP-2 and MMP-9, preventing left ventricle dysfunction and dilation. The anti-fibrotic effect induced by BM-MSCs was superior to that brought on by AT-MSCs [91], but similar benefits have been observed after intramyocardial transplantation of BM-MSCs in a model of furazolidone-induced DCM [92]. A less invasive manner to cell transplantation using intravenous infusion of BM-MSCs allowed multiple administration, which resulted in recovery of left ventricular fractional shortening and ejection fraction, which reflected in an attenuation of dilation and improved systolic function in adriamycin-induced DCM [93]. Yu and collaborators suggested that the beneficial effects of repeated BM-MSCs infusion on myocardial collagen matrix remodeling were mediated by downregulating renin–angiotensin–aldosterone system, confirmed by a rat model of doxorubicin-induced DCM [94]. This hypothesis was reinforced by the observation that multiple injections of BM-MSCs in a transgenic model of inflammatory DCM reduced inflammatory cells infiltration into the left ventricle and inhibited TNF-α/NFқBp65 signaling pathway, which was associated with reduced collagen and fibronectin deposition [95]. Interestingly, intravenous injection of BM-MSCs-derived exosomes in a murine model of doxorubicin-induced DCM has been shown to decrease circulating levels of several inflammatory factors, including TNF-α, and to reduce macrophages mobilization, which in turn prevent heart dilation and a decline in cardiac performance [96].

Recently, several studies have explored the therapeutic potential of hUC-MSCs in pre-clinical DCM models due to the advantages of having lower immunogenicity, a non-invasive source and few ethical concerns. Intramyocardial hUC-MSCs transplantation markedly improved cardiac contractile function in a transgenic mouse model of DCM by inhibiting cardiomyocytes apoptosis and preventing tissue fibrosis [97]. Cell therapy regulated the level of apoptosis-related proteins in heart tissue and increased VEGF and IGF-1, paracrine factors related to tissue repair and angiogenesis. Therapy also reduced gene expression of procollagen and collagen 1, leading to a significant reduction of fibrotic area. Similar results were observed following repeated intramuscular injections of hUC-MSCs into the limb of rats with doxorubicin-induced DCM [98]. Intravenous injection of hUC-MSCs suppress cardiac fibrosis in DCM through the control of TGF-β1/ERK-induced myocardial endothelial-mesenchymal transition (EndMT), which consists in the conversion of endothelial cells into activated fibroblasts, reducing collagen gene expression and its interstitial deposition [14,99].

Myocardial interstitial fibrosis is the most distinct histopathological hallmark of diabetic cardiomyopathy, one of the leading causes of morbidity and mortality in individuals with diabetes mellitus. This type of NICM is characterized by left ventricular hypertrophy and diastolic dysfunction, which eventually evolve to systolic dysfunction with reduced ejection fraction. MSCs transplantation markedly improves cardiac alterations in streptozotocin (STZ)-induced diabetes in rodents. BM-MSCs can migrate to myocardial tissue and an upregulated 14-3-3/p-Ask signaling pathway, reducing cardiomyocytes apoptosis in diabetic rats [100]. BM-MSCs genetically modified to express adiponectin (APN), an anti-inflammatory endocrine factor that increases insulin-sensitivity, had better antifibrotic effects than control unmodified cells, decreasing collagen expression and deposition by inhibiting TGF-β1/Smad 2/3 signaling pathway in heart from diabetic rats [101]. Similarly, BM-MSCs-derived exosomes significantly downregulated the expression of these signaling proteins with antifibrotic actions [102]. Attenuation of the pro-fibrotic FRP/Wnt/β-catenin pathway was induced by BM-MSCs preconditioned with resveratrol (RSV) resulting in enhanced cardioprotective and antifibrotic effects in diabetic cardiomyopathy [103]. Surprisingly, the most frequently prescribed antidiabetic drug, Metformin, impaired BM-MSC-mediated cardioprotection in diabetic cardiomyopathy by decreasing homing and survival of injected cells in the myocardium [104]. The efficacy of cell therapy with AT-MSCs in ameliorating myocardial inflammation and fibrosis in diabetic cardiomyopathy was explained by the macrophage polarization to the M2 phenotype in heart tissue and inhibition of the increase of IL-6 and TNF-α level, simultaneously increasing IL-10. The reduction of myocardial inflammation and injury prevented the diastolic and systolic dysfunction in diabetic rats [105,106]. Anti-inflammatory and antifibrotic effects were mediated via the production of prostaglandin E2 by AT-MSCs. Cardioprotective activity of AT-MSCs in diabetic cardiomyopathy is better achieved when cell therapy is combined with oral administration of a flavanol molecule that reduces oxidative stress, enhancing the regenerative potential of AT-MSCs [107].

Chagas disease myocarditis is the most common cause of NICM in Latin America. Chronic infection with *Trypanosoma cruzi* results in myocardial fibrosis and inflammation, leading to ventricular dilation and arrhythmias, which increase the risk of sudden death, and ultimately result in congestive HF. In advanced stages of dilated cardiomyopathy induced by Chagas disease (CCM), despite the limited homing of BM-MSCs to the heart, the intravenous injection prevented ventricular dysfunction and downregulated the expression of pro-inflammatory cytokines and the extracellular matrix protein laminin γ1 in the cardiac tissue [108]. The galectin-3 expression, a beta-galactoside-binding lectin, is crucial for survival and migration of transplanted BM-MSCs, as well as for their anti-inflammatory and anti-fibrotic effects in CCM [109]. Genetic modification of BM-MSCs to overexpress granulocyte-colony stimulating factor (G-CSF) enhanced their ability to migrate to *T. cruzi* infected hearts, becoming a more effective prevention of myocardial lesions [110]. AT-MSCs are also effective in preventing cardiac damage in CCM, because they significantly decrease the level of pro-inflammatory mediators IFN-γ, IL-6 and TNF-α in the heart while anti-inflammatory cytokine IL-10 was increased, attenuating the deposition of collagen fibers, which ultimately prevent systolic dysfunction [111].

The promising outcome of pre-clinical studies reveal the therapeutic potential of MSCs and have encouraged the clinical use of different MSC types for the treatment of diverse pathological conditions that compromise myocardial structure and function.

## 5. MSC-Based Clinical Trials for Cardiomyopathy and Coronary Disease

The promising results obtained with MSCs therapy in different pre-clinical models of cardiac diseases directed towards the elaboration of clinical research to investigate their safety and efficacy. Table 1 summarizes the list of completed and ongoing studies related to use of MSCs therapy in cardiac diseases. BM-MSCs are, so far, the most common cell type used in stem-cell based clinical trials for heart diseases. One of the pioneer’s trials using adult stem cells, the C-Cure trial, was a multicenter and randomized study that included 47 patients with chronic HF secondary to ischemic cardiomyopathy. This trial investigated the safety and efficacy of autologous BM-MSCs directly injected in the left ventricle, in which was concluded that the procedure was safe and therapy improved ejection fraction and end-systolic volume [112]. However, in the subsequent larger trial entitled CHART-1, in which 271 participants were randomized between control and treatment groups, no significant difference in primary outcome (which included all-causes mortality, HF worsening, and LVEF) was detected between the groups after 39 months of follow-up [113]. The efficacy of autologous BM-MSCs therapy ischemic cardiomyopathy was also assessed in the MESAMI pilot trial. In this open-label and single-grouped study, 10 patients with left ventricular ejection fractions (≤35%) were submitted to several intramyocardial injections, around the left ventricle, of autologous BM-MSCs, totalling 60 million cells per patient. No severe adverse event related to the procedure was registered during 24 months of follow-up, and the functional outcomes analyzed indicated an improvement in cardiac performance [114]. The MESAMI 2, a double-blinded, multicenter and placebo group trial was initiated in 2015 to validate the efficacy of this protocol, and is currently recruiting patients. The PROMETHEUS trial investigated the effects of autologous BM-MSCs therapy on cardiac structure and function in 6 patients with chronic left ventricular dysfunction secondary to myocardial infarction undergoing coronary artery bypass grafting (CABG). Cells were injected in infarcted myocardial regions not clinically able to receive bypass grafts. The fibrotic scar tissue, perfusion, heart wall thickness and contractility in injected areas were evaluated using magnetic resonance imaging (MRI) up to 18 months after cell transplantation and compared with CABG zones. MSCs-injected segments showed a significant reduction in fibrotic tissue in relation to CABG-areas. The small sample size and the absence of a control group, however, were important limitations of this trial, prematurely interrupted due to difficulties in recruitment [115]. The MSC-HF trial assessed autologous BM-MSCs therapy in a larger number of patients with severe ischemic HF and an improvement in left ventricular end-systolic volume and ejection fraction was observed in patients treated with MSCs [116]. Recently, a published trial demonstrated that autologous BM-MSCs labelled with paramagnetic particles injected in the myocardium of patients with chronic ischemic heart disease was detectable up to 14 days after transplantation [117]. Results of the recent double-blinded multi-centered CONCERT-HF trial, that analyzed the safety and efficacy of transendocardial injection of autologous BM-MSCs along with c-kit positive cardiac cells (CPCs) showed a reduced incidence of major adverse cardiac events during the 12 months of follow-up in patients with ischemic cardiomyopathy. CPCs are resident cardiac stem cells collected from patients by heart biopsies. No alteration in cardiac structure or myocardial remodeling was detected, suggesting that the beneficial effects were mediated by paracrine factors secreted by transplanted cells [118].

Clinical use of autologous MSC encounters important barriers including physiological and logistic factors, which may hinder cell harvest and expansion. The use of allogeneic, pre-prepared and standardized cell products could facilitate the translation of MSC therapy to cardiology practice. The randomized POSEIDON trial compared the safety and efficacy of autologous and allogeneic BM-MSC therapy by transendocardial injection in ischemic cardiomyopathy subjects. Patients received escalating doses (20, 100 or 200 million cells) of autologous BM-MSC or the same amount of allogeneic BM-MSC. Allogeneic BM-MSC did not induce any immune reaction or rejection. Moreover, both autologous and allogeneic cell therapy were associated with low incidence of severe adverse effects. Autologous BM-MSC, but not allogeneic, induced slight improvement in functional parameters. Both groups experienced reverse left ventricle remodeling as assessed by CT scan [120]. In the following Poseidon-DCM trial, the comparison between autologous and allogeneic BM-MSC showed allogeneic BM-MSC were associated with better outcome in ejection fraction of patients with chronic non-ischemic dilated cardiomyopathy [121]. A larger trial to investigate safety and efficacy of allogeneic BM-MSC therapy in heart failure enrolled 60 patients, randomized to receive placebo or different doses of cell therapy. Transendocardial delivery of cells was safe, and no immune response was detected in individuals submitted to cell transplantation. Despite no differences in all-causes mortality rates, post-hoc analyses suggested that patients treated with higher cell doses had reduced numbers of hospitalization and death [122]. An open-label phase IIa trial with 11 patients investigated the effects of directed injection of a novel type of MSC precursor type (iMP), isolated from allogeneic BM-MSC, in peri-infarct areas in ischemic cardiomyopathic patients during CABG. The procedure was considered safe, and the preliminary results suggested an improvement in myocardial contractility and a reduction in scar tissue after 12 months of follow-up [125]. Another trial investigated if MSC therapy could improve the left ventricle remodeling usually induced by the left ventricular assist device implantation (LVADs). Patients with advanced HF, cardiomyopathy or ventricular dysfunction received intramyocardial injection of allogeneic BM-MSC and were followed for 12 months or until heart transplantation. No significant myocarditis and myocardial rupture or adverse effects were detected, indicating that the procedure was safe [123]. The reduction of mortality rate in patients with severe heart disease was reinforced by another phase II trial [124]. In 2019, a trial was initiated (NCT03925324) which will assess the safety and possible efficacy of serial intravenous injections of allogeneic BM-MSC in cardiomyopathy patients previously implanted with LVADs. For the first time, the safety and efficacy of allogeneic BM-MSC therapy was also evaluated in cancer patients submitted to chemotherapy in the SENECA trial. In this randomized study, 37 patients with chronic anthracycline-induced cardiomyopathy were submitted to transendocardial injection of BM-MSC, which was considered a well-tolerated and safe procedure [126]. Larger trials are currently ongoing to better determine the efficacy of MSC therapy in cancer patients with HF.

The umbilical cord is another interesting source of allogeneic MSC for heart diseases therapy. UC-MSC have been shown, in vitro, to express higher levels of hepatocyte growth factor, related to myogenesis, than BM-MSC [131]. One of the first trials to assess the safety and efficacy of UC-MSC was the RIMECARD study, and intravenous infusion of UC-MSC injection in a small cohort of patients produced improvement in left ventricular ejection fraction. No differences in mortality rates, however, were detected between experimental and placebo groups after 12 months of follow-up [12]. The recently published phase I/II HUC-HEART Trial evaluated safety and efficacy of UC-MSC versus autologous BM-MSC combined with CABG in patients with chronic ischemic cardiomyopathy. After 12 months of follow-up, patients from the UC-MSC group showed an increase in left ventricular ejection fraction and a reduction in the necrotic myocardium area [127]. However, a similar randomized phase I trial using UC-MSC along with a collagen scaffold in chronic ischemic heart disease patients submitted to CABG have demonstrated that despite the absence of severe adverse effects, patients submitted to cell therapy along with the scaffold or not, had no significant change in fibrotic scar tissue [128].

Several clinical trials regarding the safety and efficacy of MSC therapy in coronary diseases have been realized or are in progress. The long-term safety and efficacy of autologous BM-MSC in patients with severe stable coronary artery disease, refractory angina and reversible myocardial ischemia was evaluated in a 3-year follow-up single-grouped trial. BM-MSC were stimulated in vitro with vascular endothelial growth-factor, in order to induce endothelial differentiation. Patients were submitted to cell injection in ischemic areas in myocardium. After 3 years, patients showed important clinical improvements, with a reduction in number of angina attacks, an increase in exercise time and quality of life parameters, in relation to baseline data prior to treatment [119]. Autologous AT-MSC therapy in refractory angina patients in the MyStromalCell Trial provide information regarding the followed-up for 3 years. During this period, functional performance in physical tests remained stable in patients treated with AT-MSC, while in the placebo group it was observed an important decline. In addition to this, an improvement in angina symptoms was registered [130]. Therefore, although several trials confirmed the safety of MSC therapy, from different tissues sources and through different delivery routes, the efficacy signs in heart diseases are still controversial and discrete. Larger phase III clinical trials are in progress, and their results will be essential to determine the perspectives of MSC therapeutic potential in cardiology.

## Figures and Tables

**Table 1:** MSC-Based Clinical Trials in Heart Diseases.

Condition	Identifier and Reference	Situation	MSCs Source	Dose	Delivery Route
Chronic Heart Failure	NCT00810238; [112] (C-Cure Trial)	Completed	Bone marrow (autologous) estimulated with a cardiogenic cocktail	600 × 10^6^ to 1200 × 10^6^ cells	Left ventricular endocardial injection
Chronic Heart Failure	NCT01768702; [113] (CHART-1 Trial)	Completed	Bone marrow (autologous) estimulated with a cardiogenic cocktail	0.6 × 10^9^ cells	Left ventricular endocardial injection
Chronic ischemic left ventricular dysfunction secondary to myocardial infarction	NCT00587990; [115] (PROMETHEUS Trial)	Terminated	Bone marrow (autologous)	N/A	Transepicardial injection
Chronic Ischemic Cardiomyopathy	NCT01076920; [114] (MESAMI Trial)	Completed	Bone marrow (autologous)	60 million cells	Intramyocardial injections
Chronic Ischemic Cardiomyopathy	NCT02462330 (MESAMI 2 Trial)	Recruiting	Bone marrow (autologous)	60 million cells	Intramyocardial injections
Ischemic Cardiomyopathy	NCT01913886	Completed	Bone marrow (autologous)	N/A	Catheterism
Coronary Heart Disease and Myocardial Ischemia	NCT00260338; [119]	Completed	Bone marrow (autologous)	Total amount of cells obtained after two passages.	Intramyocardial injection
Severe ischemic heart failure	NCT00644410; [116] (MSC-HF trial)	Completed	Bone marrow (autologous)	Total amount of cells obtained after two passages.	Intramyocardial injection
Chronic Ischemic Heart Disease	NCT03651791; [117]	Completed	Bone marrow (autologous) labelled with magnetic particles (USPIO)	10 × 10^6^ million cells	Intramyocardial injection
Ischemic Heart Disease patients pre-treated with cardiac shock wave	NCT03397095 (S-CURE Trial)	Recruiting	Bone marrow (autologous)	1 million cells/kg	Percutaneous coronary infusion
Ischemic cardiomyopathy	NCT02501811; [118] (CONCERT-HF trial	Completed	Bone marrow (autologous)	150 million cells	Transendocardial injection, combined or not with c-kit+ cells
Ischemic cardiomyopathy	NCT01087996; [120] (POSEIDON Trial)	Completed	Bone marrow (Allogeneic or autologous)	20, 100 or 200 million cells	Transendocardial injection
Chronic non-ischemic dilated cardiomyopathy	NCT01392625; [121] (POSEIDON-DCM Trial)	Completed	Bone marrow (Allogeneic or autologous)	100 million cells	Transendocardial injection
Chronic Heart Failure	NCT00721045; [122]	Completed	Bone marrow (allogeneic)	Escalating doses: 25, 75 or 150 million cells	Transendocardial injections
Advanced Heart Failure	NCT01442129; [123]	Completed	Bone marrow (allogeneic)	25 million cells	Intramyocardial injection in patients submitted to ventricular assist device (LVAD) implant
Advanced Heart Failure	NCT02362646; [124]	Completed	Bone marrow (allogeneic)	150 million cells	Intramyocardial injection in patients submitted to ventricular assist device (LVAD) implant
Heart failure Patients With Left Ventricular Assist Device	NCT03925324	Active, not recruiting	Bone marrow (allogeneic)	1.5 million cells/Kg	Three serial intravenous injection, 1 moth interval between injections
End-stage Heart Failure	NCT01759212	Active, not recruiting	Bone marrow (Allogeneic)	N/A	Intramyocardial implantation in patients submitted to ventricular assist device (LVAD) implant
Cardiomyopathy Caused by Anthracyclines	NCT02962661	Recruiting	Bone marrow (allogeneic)	N/A	Intravenous infusion (4 doses during 28 days) or transendocardial injection (15 doses)
Ischemic Cardiomyopathy	NCT01753440; [125]	Completed	Bone marrow (allogeneic)	1–4 million cells	Intramyocardial implantation during coronary artery bypass grafting
Heart Failure Caused by Anthracyclines	NCT02408432	Recruiting	Bone Marrow (allogeneic)	N/A	Intravenous infusion once weekly for 4 weeks
Congenital Heart Disease	NCT04236479 (MedCaP Trial)	Recruiting	Bone Marrow (allogeneic)	Escalating doses from 1 to 80 x10^6^ cells/Kg	Cardiopulmonary bypass
Left ventricular dysfunction secondary to anthracycline-induced cardiomyopathy	NCT02509156; [126] (SENECA Trial)	Completed	Bone marrow (Allogeneic)	100 million cells	Transendocardial injection
Ischemic Heart Disease in type 2 diabetic patients	NCT04776239 (ACESO-IHD Trial)	Not yet recruiting	Bone marrow (Allogeneic)	100 million cells	Intravenous injection
Non-Ischemic Dilated Cardiomyopathy	NCT04476901	Recruiting	Allogeneic	80–100 million cells	Transendocardial Injection
Chronic Ischemic Cardiomyopathy	NCT02323477; [127] (HUC-HEART Trial)	Terminated	Umbilical cord or Bone marrow (autologous)	23×10^6^ 70×10^7^	Intramyocardial injection
Chronic Ischemic Cardiomyopathy	NCT02635464; [128]	Completed	Umbilical cord	1 × 10^8^ cells	Injection in infarct region, along with collagen scaffold or not
Ischemic Cardiomyopathy	NCT02368587	Not yet recruiting	Umbilical cord (Wharton’s jelly)	N/A	Intracoronary or Intravenous Infusion
Chronic Heart Failure of Non-ischemic Etiology	NCT04325594 (RegenHeart Trial)	Enrolling by invitation	Umbilical cord	10 million cells	Intracoronary administration
Heart Failure	NCT01739777; [12] (RIMECARD Trial)	Completed	Umbilical cord	1 × 10^6^ cells/kg	Intravenous infusion
Heart Failure and Coronary Disease	NCT04011059	Not yet recruiting	Umbilical cord (Wharton’s jelly)	N/A	Placement of an extracellular matrix patch cells cultured on the epicardial surface and cell injection around the infarcted zone
Coronary Artery Disease	NCT04551456	Not yet recruiting	Umbilical cord (Wharton’s jelly)	1 to 3 doses of 1 × 10^6^ cells/kg	Intravenous infusion
Ischemic Heart Disease and Heart Failure	NCT02387723, [129]	Completed	Adipose tissue (allogeneic)	100 million cells	Intramyocardial injection
Refractory Angina and Coronary Artery Stenosis	NCT01449032; [130] (MyStromalCell Trial)	Completed	Adipose tissue (autologous)	Total amount of cells obtained after two passages.	Intramyocardial injection
Ischemic Cardiomyopathy	NCT04695522	Recruiting	Adipose tissue	N/A	Cell spray in fibrinogen and thrombin solutions
Ischemic Heart Disease and Coronary Artery Disease	NCT04005989 (ADMIRE Trial)	Not yet recruiting	Adipose tissue (autologous)	Low dose arm: 1 × 10^6^ cells/kg Intermediate dose arm: 2 × 10^6^ cells/kg High dose arm: 4 × 10^6^ cells/kg	Intramyocardial injection
